# Mesenchymal stem cells are sensitive to bleomycin treatment

**DOI:** 10.1038/srep26645

**Published:** 2016-05-24

**Authors:** Nils H. Nicolay, Alexander Rühle, Ramon Lopez Perez, Thuy Trinh, Sonevisay Sisombath, Klaus-Josef Weber, Anthony D. Ho, Jürgen Debus, Rainer Saffrich, Peter E. Huber

**Affiliations:** 1 Department of Radiation Oncology, Heidelberg University Hospital, Im Neuenheimer Feld 400, 69120 Heidelberg, Germany; 2 Heidelberg Institute for Radiation Oncology (HIRO), National Center for Radiation Research in Oncology, Im Neuenheimer Feld 280, 69120 Heidelberg, Germany; 3 Department of Molecular and Radiation Oncology, German Cancer Research Center (dkfz), Im Neuenheimer Feld 280, 69120 Heidelberg, Germany; 4 Department of Hematology and Oncology, Heidelberg University Hospital, Im Neuenheimer Feld 410, 69120 Heidelberg, Germany

## Abstract

Mesenchymal stem cells (MSCs) have been shown to attenuate pulmonary damage induced by bleomycin-based anticancer treatments, but the influence of bleomycin on the stem cells themselves remains largely unknown. Here, we demonstrate that human bone marrow-derived MSCs are relatively sensitive to bleomycin exposure compared to adult fibroblasts. MSCs revealed increased levels of apoptosis after bleomycin treatment, while cellular morphology, stem cell surface marker expression and the ability for adhesion and migration remained unchanged. Bleomycin treatment also resulted in a reduced adipogenic differentiation potential of these stem cells. MSCs were found to efficiently repair DNA double strand breaks induced by bleomycin, mostly through non-homologous end joining repair. Low mRNA and protein expression levels of the inactivating enzyme bleomycin hydrolase were detected in MSCs that may contribute to the observed bleomycin-sensitive phenotype of these cells. The sensitivity of MSCs against bleomycin needs to be taken into consideration for ongoing and future treatment protocols investigating these stem cells as a potential treatment option for bleomycin-induced pulmonary damage in the clinic.

The anti-cancer agent bleomycin was first purified from Streptomyces verticillus cultures and gained approval by the United States Food and Drug Administration in 1973^1^; the licensed drug is mainly comprised of two glycopeptide derivatives of the bleomycin family, called bleomycins A2 and B2^2^. Bleomycin-based treatment regimens have been successfully used for a variety of different cancers, including Hodgkin’s lymphoma, testicular, ovarian and cervical cancers^3^,^4^,^5^. Bleomycin acts by creating DNA double strand breaks, mimicking the effects of ionizing radiation, but the underlying exact mechanisms of action remain incompletely understood^6^. It has been suggested that bleomycin forms complexes with iron ions and thereby forms a pseudoenzyme that can create hydroxide and superoxide radicals that in turn induce DNA strand breaks^7^. Additionally, bleomycin has been shown to mediate oxidation of lipids and other cellular molecules^8^. The clinical use of bleomycin is commonly limited by the drug’s toxicity to the lung, and irreversible bleomycin-induced pulmonary damage and fibrosis have been described to occur in up to one fifth of all patients^9^.

Mesenchymal stem cells (MSCs) were first identified in bone marrow samples, but can be isolated from various other tissues, including vascular and adipose tissues, kidney, skin and umbilical cord^10^,^11^. Unlike their hematopoietic counterparts, MSCs comprise a heterogeneous group of multipotent stromal cells. As no unique set of MSC surface markers is generally accepted, characterization of these stem cells depends on morphological and functional criteria, like the cells’ fibroblast-like spindle shape, their ability to adhere to plastic surfaces and migrate and there potential for differentiation along the adipogenic, chondrogenic and osteogenic lineages^12^,^13^. Due to their differentiation capability, MSCs have come into focus as a potential means of treating tissue damage. MSC-based therapies have been shown to attenuate various forms of organ damage in preclinical models, both by differentiating into tissue-specific functional cells and by creating a regenerative microenvironment^14^,^15^. Beneficial effects of MSCs on bleomycin-induced pulmonary damage have been demonstrated in animal models: MSC infusions were found to improve pulmonary function, attenuate histopathological markers of lung fibrosis and increase animal survival after bleomycin treatment^16^,^17^. However, the effects of bleomycin on the MSCs themselves remain largely unknown.

Here, we characterized the influence of bleomycin treatment on the viability, proliferation and functional abilities of MSCs. We further investigated the bleomycin-induced effects on the defining stem cell properties and surface marker expression of these stem cells.

## Results

### MSCs are sensitive to bleomycin treatment

**Figure 1:**
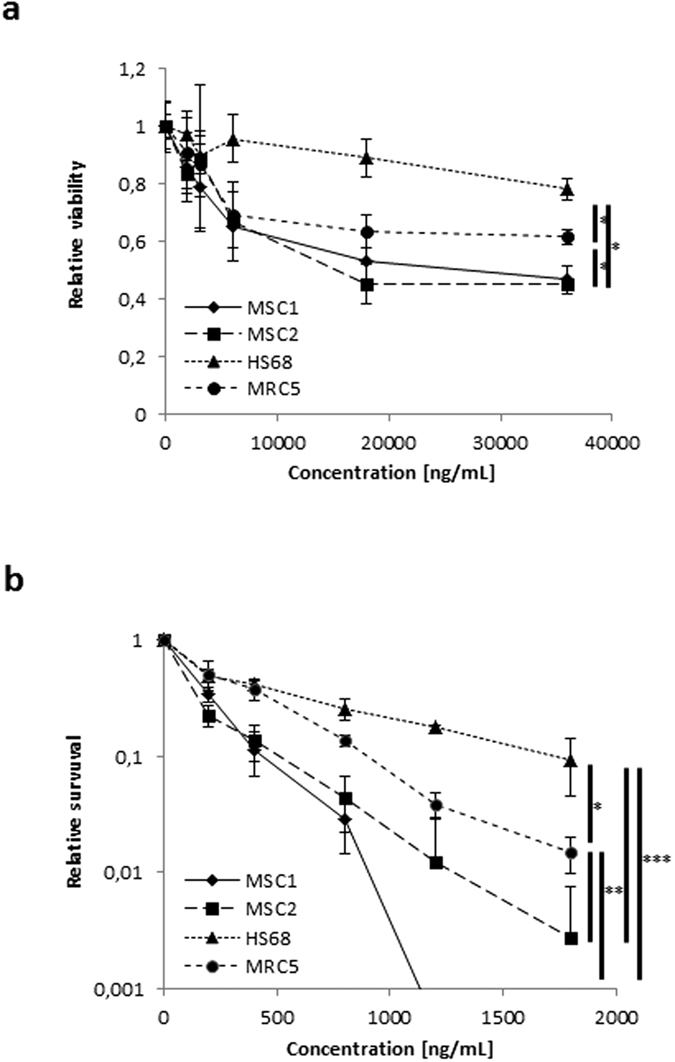
MSCs show reduced viability and survival compared to adult fibroblasts after bleomycin treatment. (**a**) MTS assay data showing viability of two different MSCs and two adult fibroblast cell lines after treatment with bleomycin. (**b**) Clonogenic survival assays for MSCs and fibroblasts after bleomycin treatment. Data are mean +/− SD (n = 3). *P < 0.05, **P < 0.01, ***P < 0.001 (Student’s t-test).

Bleomycin sensitivity of human MSCs and differentiated fibroblast cell lines HS68 and MRC5 was investigated by viability and clonogenic survival assays; the treatment doses and exposure times used in our experiments were chosen to mimic the conditions of patients undergoing bleomycin chemotherapy. After treatment with different concentrations of bleomycin, both tested MSC samples demonstrated considerably reduced viability compared to HS68 fibroblasts and a moderate reduction in viability compared to MRC5 cells (*P* < 0.05, two-sided Student’s t-test) (Fig. 1a).

Furthermore, clonogenic survival of MSC1 and MSC2 specimens was found to be strongly reduced when compared to both tested fibroblast cell lines after 4-hour treatment with doses up to 1800 ng/mL bleomycin (*P* < 0.001 for HS68, *P *< 0.01 for MRC5) (Fig. 1b).

### MSCs demonstrate increased apoptosis after bleomycin exposure

**Figure 2:**
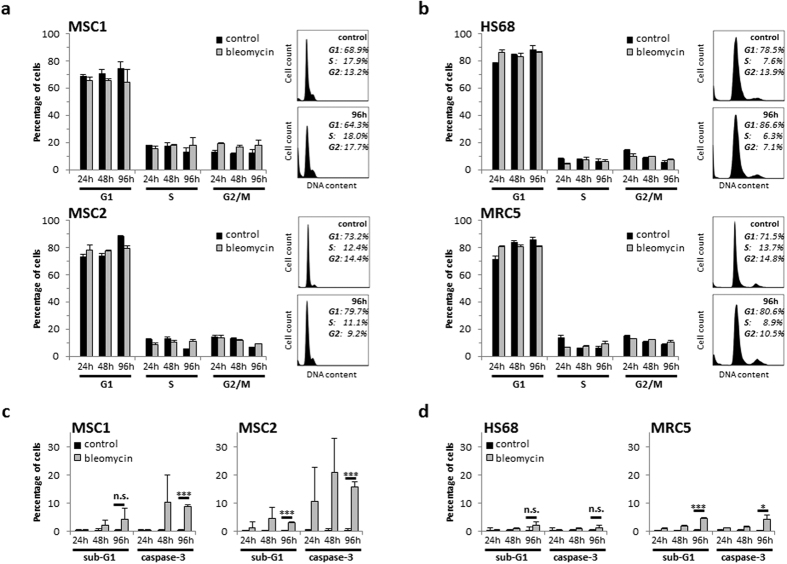
Bleomycin treatment results in increased apoptosis but no cell cycle changes in MSCs. Cell cycle distribution of two MSC samples (**a**) and two adult fibroblast cell lines (**b**) after 4-hour exposure to 1800 ng/mL bleomycin. (**c**,**d**) Percentage of apoptotic MSCs and adult fibroblasts after treatment with 1800 ng/mL bleomycin as assessed by sub-G1 population and caspase-3 activation. Data are mean +/− SD (n = 3). *P < 0.05, ***P < 0.001.

Cell cycle profiles of MSCs and fibroblasts after treatment with bleomycin were assessed by FACS analyses. Exposure to 1800 ng/mL bleomycin for 4 hours did not result in general changes of cell cycle distribution in the MSC samples or human fibroblast cell lines (Fig. 2a,b). Of note, there was no accumulation in G2 phase, as reported previously for other DNA double strand break-inducing agents in MSCs^18^,^19^.

Apoptosis induced by bleomycin was measured both by cellular sub-G1 population and activation of caspase-3. At 96 hours after treatment, both MSC1 and MSC2 showed increased levels of apoptosis as measured by caspase-3 activation (*P* < 0.001); while both MSC specimens also demonstrated increases of the sub-G1 populations, this increase reached significance only for MSC2 (*P* < 0.001), but not for MSC1 cells (*P* = 0.11) (Fig. 2c). Similarly, MRC5 lung fibroblasts demonstrated a significant, albeit comparatively lesser increase in apoptotic cells after bleomycin treatment (*P* < 0.001 for sub-G1 analyses, *P* < 0.05 for caspase-3 activation). In contrast, HS68 fibroblasts did not exhibit increased levels of apoptosis after exposure to bleomycin (Fig. 2d).

### Bleomycin does not affect MSC adhesion and migration

**Figure 3:**
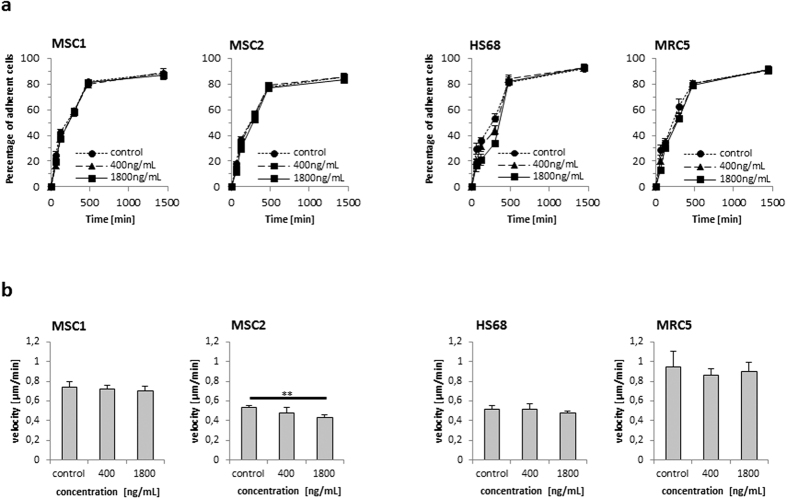
Bleomycin treatment does not affect the ability of MSCs for adhesion and migration. (**a**) Relative adhesion rates of MSCs and differentiated fibroblasts up to 24 hours after 4-hour treatment with 400 or 1800 ng/mL bleomycin (n = 5). (**b**) Average velocity of MSCs and differentiated fibroblasts after treatment with 400 and 1800 ng/mL bleomycin. Data are mean +/− SD. **P < 0.01.

The adherence potential of MSCs is a defining hallmark of these cells and is commonly used as a first isolation step. MSC adhesion on plastic surfaces was investigated up to 24 hours after exposure to 400 or 1800 ng/mL bleoymcin for 4 hours. Generally, the adherence potential of MSCs was found to be largely unaffected by bleomycin treatment, and there was no significant delay in adhesion caused by bleomycin (Fig. 3a). At 24 hours, attachment levels of MSC1 and MSC2 cells were well above 80% with no significant difference between untreated and treated specimens (88.4% vs. 89.0% vs. 86.8% for MSC1, n.s.; 85.2% vs. 85.6% vs. 83.2% for MSC2, n.s.). Both tested adult fibroblast cell lines reached similar levels of attachment at 24 hours independent of bleomycin treatment (92.0% vs. 93.0% vs. 92.6% for HS68, n.s.; 91.0% vs. 90.4% vs. 91.4% for MRC5, n.s.).

The migratory ability of MSCs and adult fibroblasts after bleomycin exposure was measured by time-lapse microscopy. While MSC1 cells did not show a reduction in cellular movements, a small but significant reduction of average velocity was observed in MSC2 samples after treatment with high doses of 1800 ng/mL bleomycin (*P* < 0.01). The average velocity of HS68 and MRC5 adult fibroblast cell lines remained unaffected by low or high doses of the anticancer drug (Fig. 3b).

### Bleomycin does not alter MSC surface marker expression

**Figure 4:**
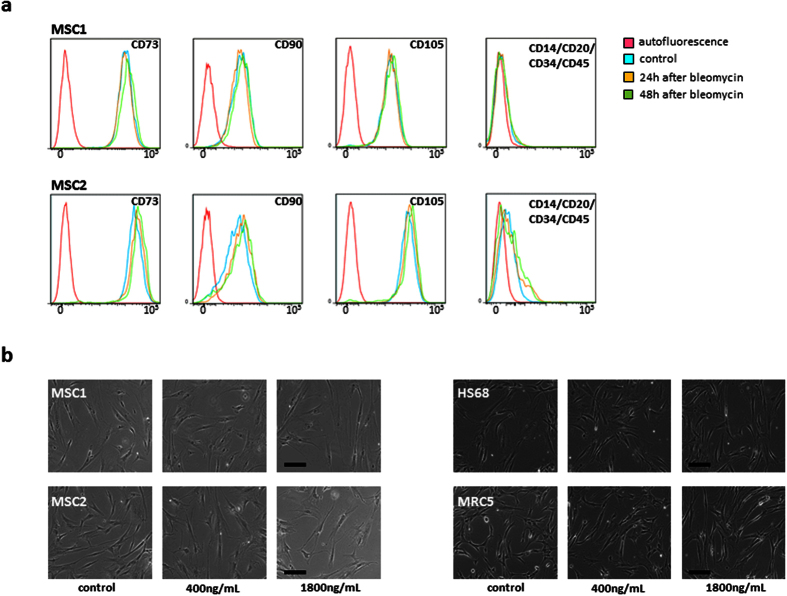
MSCs stably express defining surface markers after bleomycin treatment. (**a**) Representative FACS analyses of defining positive MSC markers CD73, CD90 and CD105 and negative markers CD14, CD20, CD34 and CD45 at 24 and 48 hours after treatment with 1800 ng/mL bleomycin. (**b**) Representative microscopic images of unstained MSCs and adult fibroblasts showing no measurable changes in morphology after treatment with 400 or 1800 ng/mL bleomycin (20× objective, scale bar 100 μm).

Expression patterns of MSC surface markers were analyzed by FACS analysis at 24 and 48 hours after treatment with 1800 ng/mL bleomycin. Expression of positive stem cell markers CD73, CD90 and CD 105 were stably expressed in both MSC1 and MSC2 after exposure to high doses of bleomycin (Fig. 4a). Additionally, negative surface markers CD14, CD20, CD34 and CD45 as defined by the International Society for Cell Therapy^12^ were found unaffected at 24 and 48 hours after bleomycin treatment.

Cellular morphology of MSCs and adult fibroblasts remained largely identical after exposure to 400 and 1800 ng/mL bleomycin, and no loss of spindle shape or signs of increased apoptosis could be detected by light microscopy at 24 hours after bleomycin treatment (Fig. 4b).

### Bleomycin reduces adipogenic, but not chondrogenic differentiation potential of MSCs

**Figure 5:**
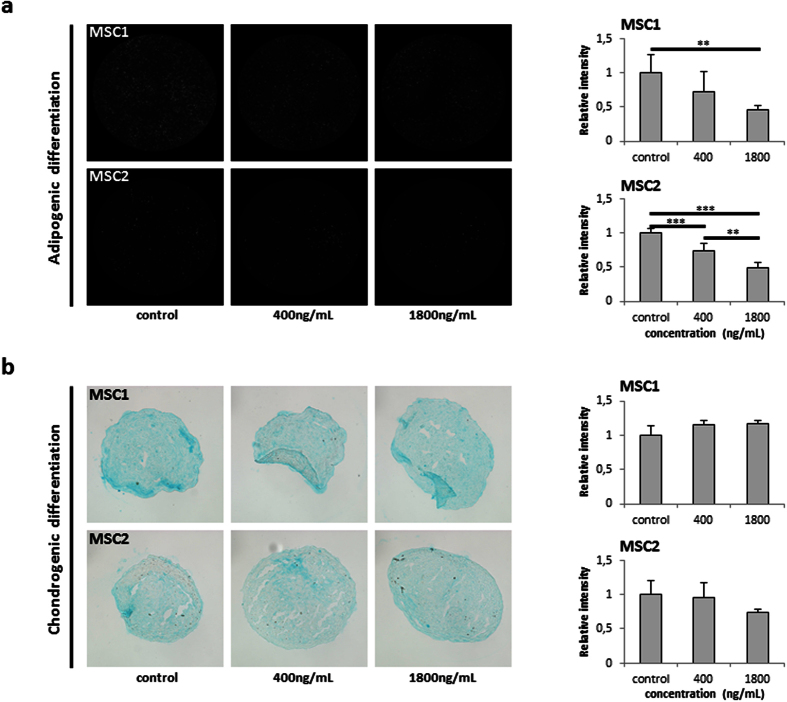
Bleomycin treatment reduces adipogenic but not chondrogenic differentiation potential of MSCs. (**a**) Representative BODIPY lipid staining of MSC1 and MSC2 samples after 4-hour exposure to 400 and 1800 ng/mL bleomycin to assess adipogenic differentiation. (**b**) Representative Alcian blue staining for chondrogenic differentiation in MSC1 and MSC2 samples after bleomycin treatment. Relative staining intensities were measured to quantify differentiation levels after adipogenic and chondrogenic differentiation. Data are mean +/− SD. **P < 0.01, ***P < 0.001.

MSCs are characterized by their ability to differentiate along the adipogenic and chondrogenic lineages. A potential influence of bleomycin treatment on the differentiation potential of human MSCs was assessed by immunocytochemical analyses after induced differentiation. 4-hour exposure to bleomycin resulted in a reduced adipogenic differentiation of both MSC samples in a dose-dependent manner (Fig. 5a). While MSC1 revealed a non-significant reduction after 400 ng/mL bleomycin (*P* = 0.12), treatment with 1800 ng/mL strongly reduced adipogenic differentiation (*P* < 0.01). Exposure of MSC2 cells to both low and high concentrations of bleomycin led to a decrease in induced adipogenic differentiation (*P* < 0.001 for 400 and 1800 ng/mL).

In contrast, bleomycin treatment did not significantly alter the ability of MSCs to differentiate along the chondrogenic lineage, and the differentiation potential of both tested MSC specimens was preserved even after exposure to 1800 ng/mL bleomycin (*P* = 0.19 for MSC1, *P* = 0.11 for MSC2) (Fig. 5b).

### MSCs efficiently repair bleomycin-induced DNA double strand breaks

**Figure 6:**
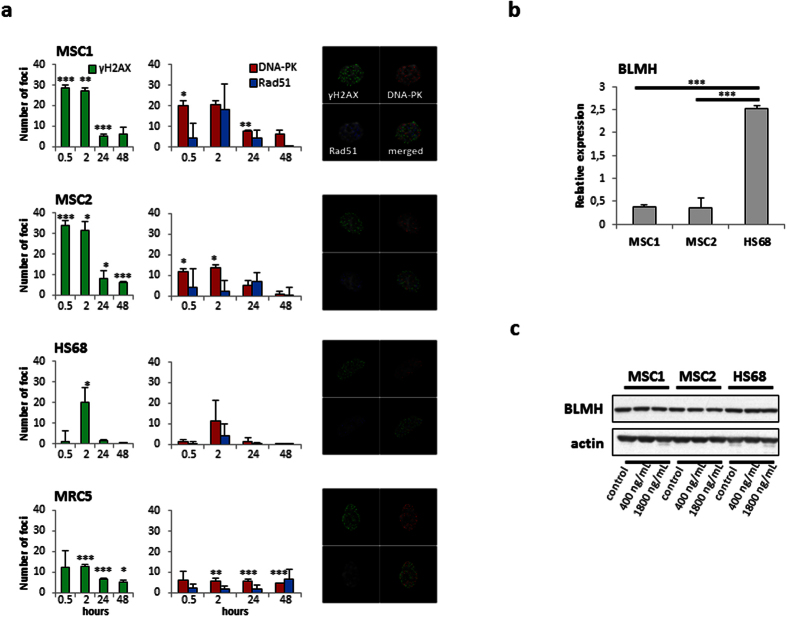
High bleomycin sensitivity of MSCs is associated with low BLMH expression, but not associated with inefficient DNA double strand break repair. (**a**) Number of γH2AX foci (left panel) and DNA-PK and Rad51 foci (right panel) in MSCs and adult fibroblasts at various time points after treatment with 1800 ng/mL bleomycin for 4 hours. Representative sample pictures of nuclei were taken at 400× magnification. (**b**) Relative mRNA expression of the BLMH gene in MSCs and HS68 fibroblasts. (**c**) Western blot analyses showing BLMH protein expression in MSCs and HS68 fibroblasts at 24 hours after treatment with 400 or 1800 ng/mL bleomycin. Data are mean +/− SD. *P < 0.05, **P < 0.01, ***P < 0.001.

The ability of MSCs to repair DNA double strand breaks induced by bleomycin was investigated as a potential mechanism causing the observed bleomycin sensitivity of these stem cells. Immunofluorescence analyses of γH2AX foci as markers of DNA double strand breaks (DSBs) demonstrated high numbers of initial strand breaks at 30 minutes and 2 hours after completion of a 4-hour bleomycin treatment in both MSC samples; DSB numbers in both adult fibroblast cell lines were found considerably lower at these time points (Fig. 6a). Levels of residual γH2AX foci were still significantly elevated at 24 hours in MSC1 and MSC2 cells treated with bleomycin (*P* < 0.001 for MSC1, *P* < 0.05 for MSC2) and remained significantly increased up to 48 hours in MSC2 (*P* < 0.001). In bleomycin-resistant HS68 fibroblasts, γH2AX levels returned to baseline at 24 hours, while MRC5 cells revealed slow repair kinetics with increased residual γH2AX numbers at both 24 and 48 hours (*P* < 0.001 and *P* < 0.05 for 24 and 48 hours, respectively).

Foci of the DNA protein kinase (DNA-PK) and Rad51 were investigated to assess the influence of the two main DNA double strand break repair mechanisms, the non-homologous end joining pathway and the homologous recombination repair pathway, respectively (Fig. 6b). Numbers of DNA-PK foci were found increased in both MSC samples at 30 min after bleomycin treatment (*P *< 0.05); while numbers remained elevated up to 24 hours, this elevation only reached statistical significance in MSC1 samples (*P* < 0.01 for MSC1, *P* = 0.11 for MSC2). In adult HS68 fibroblasts, DNA-PK foci showed a small, non-significant increase at 2 hours (*P* = 0.13) and returned to baseline levels at 24 hours. In MRC5 lung fibroblasts, bleomycin treatment did not result in an initial formation of DNA-PK foci at 30 min, while foci numbers were found slightly, but significantly increased at 2, 24 and 48 hours (*P* < 0.01 at 2 hours, *P* < 0.001 at 24 and 48 hours).

Rad51 foci were found to be not significantly increased after 4-hour treatment with bleomycin in either MSC population, although there was a non-significant elevation at 2 hours in MSC1 samples (*P* = 0.20). Similarly, both tested adult fibroblast cell lines did not reveal an increase in Rad51 foci following exposure to bleomycin, suggesting that homologous recombination repair is not strongly involved in the removal of bleomycin-induced DNA double strand breaks in either MSCs or fibroblasts.

### MSCs express lower levels of inactivating enzyme bleomycin hydrolase

Expression of the cysteine peptidase, bleomycin hydrolase (BLMH) has been linked to the ability of cells to hydrolyze and thereby efficiently inactivate intracellular bleomycin. Gene array data obtained from MSC1 and MSC2 samples demonstrated about 7-fold lower expression levels of BLMH mRNA compared to the relatively resistant HS68 adult fibroblasts (*P* < 0.001 for both MSCs) (Fig. 6b). Likewise, HS68 demonstrated about 1.6-fold higher expression of BLMH protein than both tested MSCs (Fig. 6c), although the differences in BLMH protein expression were less pronounced between MSCs and HS68 fibroblasts. Interestingly, there was no marked regulation of BLMH protein expression at 24 hours after treatment with 400 or 1800 ng/mL bleomycin in either cell type.

## Discussion

While various publications have demonstrated that MSCs may exert beneficial effects on tissue lesions caused by bleomycin treatment, the influence of this cytotoxic drug on the stem cells themselves remains widely unknown^16^,^20^,^21^. Here, we demonstrated that human MSCs were relatively sensitive to bleomycin treatment in comparison to adult fibroblasts as measured by cellular viability and clonogenic colony formation. Bleomycin concentrations and treatment times used in our experiments were chosen to mimic the conditions observed in patients receiving bleomycin-based therapy, where peak plasma concentrations between 130 and 5000 ng/mL and a plasma half-life below 2 hours have been reported^22^,^23^.

Several analyses have so far provided inconsistent results regarding the sensitivity of MSCs against various chemotherapeutic agents like etoposide, vincristine, cisplatin, kinase inhibitors or ionizing radiation^19^,^24^,^25^,^26^,^27^,^28^. A relative *in-vitro* resistance of MSCs against vincristine, cisplatin or etoposide has been demonstrated in cell culture experiments, and bone marrow samples of patients treated with these drugs have been shown to harbor viable and proliferating MSCs, confirming the observed resistant phenotype *in vivo*^29^,^30^. Most of the investigated drugs exert their effect by creating various forms of DNA damage, effectively hampering DNA replication and transcription^31^. In line with the described resistance of MSCs against different DNA damaging agents, it has been suggested that these stem cells have the potential to efficiently repair potentially lethal DNA lesions, especially DNA double strand breaks^32^. While we demonstrated that MSCs were able to repair the majority of DNA double strand breaks induced by bleomycin within 24 hours, MSCs were still found to be sensitive to the DNA double strand break-inducing agent bleomycin in our dataset. In line with the observed sensitivity, bleomycin-treated MSCs demonstrated significant albeit small increases in apoptosis at late time points. Previous publications suggested that MSCs were relatively resistant against apoptotic activation upon induction of DNA damage, likely due to a reduced p73-dependent activation of pro-apoptotic factors and high constitutive expression levels of anti-apoptotic proteins such as Bcl-2 and Bcl-xL^18^,^33^. Unlike most other DNA-damaging cytotoxic drugs, bleomycin exerts its effects by creating highly reactive oxygen radicals, and it has been suggested that reactive oxygen species could result in the induction of apoptosis in MSCs^34^,^35^.

The cellular response to bleomycin has been strongly linked to the expression of the neutral cysteine protease, bleomycin hydrolase (BLMH); high expression levels of this inactivating enzyme have been shown to protect against bleomycin-induced cell damage both *in vitro* and in animal models^36^,^37^,^38^. Differential expression of BLMH has been reported for different normal tissues and cancer cell lines, and functional analyses suggest that relatively small changes in the expression of BLMH had considerable effects regarding the cellular sensitivity to bleomycin treatment^22^,^39^. In our dataset, both tested MSC samples demonstrated lower mRNA and protein expression of the bleomycin-inactivating enzyme, BLMH compared to the relatively resistant HS68 fibroblast cell line. While the difference was considerably more pronounced on the mRNA level than on the protein level, this differential expression may still at least in part be able to explain the observed bleomycin sensitivity of MSCs compared to adult fibroblasts.

Beyond the bleomycin-sensitive phenotype of human MSCs, we demonstrated that bleomycin had only limited influence on the characteristic stem cell properties of these cells. The potential for adherence to plastic surfaces is a defining feature of MSCs and is commonly used to select these cells in culture^12^. The ability to adhere was found to be unaffected even by high doses of bleomycin, and there was no delay in adherence for either MSC sample when compared to untreated control cells. In accordance with these findings, various other cytotoxic drugs have been reported to only marginally influence MSC adherence, and upregulation of different genes involved in cellular adhesion has been reported in these stem cells^19^,^40^.

Similarly, the ability to differentiate along several lineages has been established as a defining feature of MSCs; this ability is believed to be the basis for the reported regenerative potential of these stem cells^41^. This dataset revealed for the first time that bleomycin treatment resulted in a reduced ability to differentiate along the adipogenic lineage in a dose-dependent manner, while the potential for chondrogenic differentiation was preserved even after high doses of bleomycin. Cell culture data have suggested that the loss of the defining differentiation potential of MSCs occurs in a hierarchical manner, whereby the ability for adipogenic differentiation is commonly abolished first prior to the loss of the chondrogenic differentiation potential, finally giving rise to osteogenic precursor cells^42^,^43^. The observed reduction in the differentiation potential of MSCs may bear therapeutic significance, as previous publications have reported regenerative effects of mobilized endogenous MSCs in bleomycin-induced pulmonary fibrosis^44^,^45^. In these datasets, pulmonary damage was mostly induced by topical intratracheal instillation of bleomycin, thereby preserving MSCs of other tissues^46^. However, the translation of these results into the clinic may be strongly hampered by the fact that systemic administration of bleomycin may also damage distant bleomycin-sensitive MSCs, thereby preventing a recruitment of these cells to the pulmonary lesion sites. Our findings regarding a high sensitivity of MSCs to bleomycin may also provide a potential explanation by the drug’s robust ability to promptly induce lung damage and fibrosis, both due to the primary toxic insult of the chemotherapeutic agent and by disabling the protective function of pulmonary MSCs.

While there is a strong need for a causative therapy to treat pulmonary damage induced by bleomycin, no such therapy has been established to date. MSC infusions have been widely studied in this context, as their ability for differentiation and creation of a protective microenvironment has made these cells an attractive target for tissue regeneration. It has been demonstrated that MSCs could differentiate into type II alveolar epithelial cells following bleomycin-induced lung injuries, effectively reducing oxidative stress in the damaged tissue^47^. Similarly, it has been suggested that transplanted MSCs could strongly suppress the inflammatory response of pulmonary tissue to intratracheal instillation of bleomycin, and reduced pulmonary and serum levels of matrix metalloproteinase-9, tissue inhibitor of metalloproteinase-1, interferon-γ and transforming growth factor-β have been observed following MSC treatment for bleomycin-induced lung damage^48^,^49^. Hypoxia could further augment the protective paracrine effects of MSCs in lung tissue, as it resulted in an additional upregulation of anti-apoptotic and anti-inflammatory mediators and growth factors^21^. However, the vast majority of studies have focused on the effects of MSCs on the initial inflammatory phases of lung damage after bleomycin treatment, and little is known about the influence of the stem cells on the later fibrotic stages^20^.

Most publications studying the therapeutic implications of MSC-based treatments utilized exogenous stem cell infusions. While the reported bleomycin-sensitive phenotype of MSCs may not be directly relevant to these treatments, bystander effects have been described for bleomycin-naïve MSCs *in vitro* and may also affect exogenous MSCs after integration into bleomycin-damaged lung tissue, depending on the MSC treatment schedule^50^. Additionally, potential adverse effects of exogenous MSC transplantations remain incompletely studied, including the role of the stem cells’ potential carcinogenic and tumor-protecting effects in the context of anti-cancer treatments^14^,^51^. While the first clinical trials testing allogeneic MSC-based treatment options for other forms of pulmonary fibrosis are currently underway, utilizing endogenous MSCs for the treatment of lung damage may help to avoid some of the problems connected with allogeneic MSC transplantations^52^,^53^. It has been shown that re-transplantation of resident pulmonary mesenchymal progenitor cells could more potently reduce bleomycin-induced pulmonary inflammation and fibrotic thickening than exogenous adipose-derived MSCs^54^. Nevertheless, based on our findings, the observed bleomycin-sensitive phenotype of these stem cells will have to be taken into consideration when devising MSC-based treatment protocols for pulmonary fibrosis. In line with our findings, it has been reported that the induction of bleomycin-induced lung damage was associated with the loss of resident pulmonary MSCs, suggesting that this form of pulmonary injury may at least partly be due to the loss of the protective functions of bleomycin-sensitive MSCs^55^. Additional *in-vivo* data are needed to further clarify the exact role of bleomycin-sensitive MSCs in the context of bleomycin-induced pulmonary injury.

In summary, the data presented here demonstrated that MSCs were relatively sensitive to bleomycin exposure, and revealed a reduction in their adipogenic differentiation potential while maintaining the majority of their defining stem cell characteristics. The bleomycin-sensitive phenotype of MSCs may at least be partly responsible for the robust induction of pulmonary damage after treatment with this chemotherapeutic drug.

## Materials and Methods

### Cell culture

Primary human MSC1 and MSC2 mesenchymal stem cell preparations were obtained from healthy voluntary donors and isolated as previously described^56^. MSCs were grown in Mesenchymal Stem Cell Growth Medium (*MSCGM*^*TM*^, Lonza, Basel, Switzerland), supplemented with *MSCGM*^*TM*^ SingleQuots (Lonza) and were maintained in a humidified incubator at 37 °C and 5% CO_2_. HS68 human dermal fibroblasts were purchased from the ATCC (Manassas, USA) and were cultured in Dulbecco’s Modified Eagle Medium (Biochrom, Berlin, Germany), supplemented with 10% fetal bovine serum and 3.5 g/L glucose. MRC5 human pulmonary fibroblasts were received from the ATCC and grown in Eagle’s Minimum Essential Medium (Biochrom); 10% fetal bovine serum was added to the culturing medium. Written informed consent was obtained from the donors prior to MSC harvesting; the study was approved by the independent ethics board of the University of Heidelberg, and all experiments were carried out in accordance with the approved guidelines.

### Drug preparation

Bleomycin stock solution was purchased from the central pharmacy of Heidelberg University Hospital and was kept refrigerated for up to 7 days. The drug was diluted in culturing medium to the required concentrations prior to carrying out each experiment. All experimental setups were protected from light after addition of bleomycin.

### Viability assays

Cellular viability after bleomycin treatment was measured using the MTS assay. 2 000 cells were grown in each well of a 96-well plate, and cells were incubated with bleomycin at concentrations between 1800 and 36 000 ng/mL for 5 days. 20 μL of 1.9 mg/mL MTS reagent (Promega, Madison, USA) was added to each well for 2 hours, and absorbance at 490 nm was then measured using a microplate reader (Tecan, Crailsheim, Germany).

### Clonogenic survival assays

Cells were plated 6 hours prior to treatment to enable attachment. Bleomycin was added at concentrations between 200 and 1800 ng/mL for 4 hours before medium was replaced. Cells were maintained for a further 14 days to allow cells to form colonies. Colonies were fixed with 25% acetic acid (v/v) in methanol prior to staining with crystal violet solution. Colonies with more than 50 cells were then counted using a light microscope. All clonogenic assay experiments were performed in triplicate. The surviving fraction of cells was calculated according to the following formula: (#colonies/#plated cells)_treated_/(#colonies/#plated cells)_untreated_.

### Cell cycle and apoptosis measurements

To analyze cell cycle profiles and levels of apoptosis, cells were treated with 1800 ng/mL bleomycin for 4 hours before replacement of medium. At the time points indicated below, cells were harvested and fixed in 4% paraformaldehyde solution before resuspension in ice-cold 70% ethanol. Cells were then centrifuged and incubated with 1 μg/mL 4′,6-diamidin-2-phenylindol (DAPI) solution. For apoptosis measurements, cells were washed in PBS. After further centrifugation, a fluorescence-coupled antibody against activated caspase-3 (1:20, BD Pharmingen, Heidelberg, Germany) was added for 1 hour at room temperature. Flow cytometry analyses were performed on a LSR II system (Becton-Dickinson, Heidelberg, Germany), and 10 000 events were counted for each experimental condition. Cell cycle profile modeling was carried out using FlowJo 7.6.5 software (FlowJo LLC, Ashland, USA).

### Adhesion measurements

Cells were exposed to 400 or 1800 ng/mL bleomycin for 4 hours before medium was exchanged. 100 cells were then added to each well of a 96-well plate, and attached cells were counted using a light microscope over a time period of 24 hours. The attachment efficiency was calculated as the ratio between attached and plated cells. All cell adhesion measurements were repeated at least three times.

### Migration measurements

Cells were plated in 24-well plates to a confluence of below 50% and treatment with 400 or 1800 ng/mL bleomycin was performed for 4 hours. Cellular migration was then measured every 7 minutes over a time period of 35 hours using time-lapse imaging. Microscopy was carried out on an IX70 inverted microscope fitted with an incubator box (Olympus, Hamburg, Germany). Cell migration was quantified by manual single-cell tracking using ImageJ software (National Institutes of Health, Bethesda, USA).

### Surface marker measurements

MSCs were grown up to 80% confluency before treatment with 1800 ng/mL bleomycin for 4 hours. 24 and 48 hours later, cells were harvested, and expression levels of positive and negative surface markers were examined as proposed as minimal criteria by the International Society of Cell Therapy^12^. Surface marker stainings were performed using the MSC Phenotyping Kit (Miltenyi Biotec, Bergisch-Gladbach, Germany) according to the manufacturer’s instructions. Measurements were carried out on a FACSCanto flow cytometer (Becton-Dickinson, Heidelberg, Germany) followed by data analysis using FlowJo 7.6.5 software.

### Quantification of MSC differentiation

Differentiation experiments were carried out on log phase MSCs plated in 24-well plates. Stem cells were treated with 400 or 1800 ng/mL bleomycin for 4 hours before replacement of medium. At 24 hours after treatment, medium was exchanged for differentiation media, and cells were proliferated for further 21 days. All differentiation media were replaced twice per week. StemPro® Adipogenesis Differentiation Kit (Gibco Life Technologies, Frankfurt, Germany) was used according to the manufacturer’s instructions to induce adipogenic differentiation. Adipogenic differentiation was assessed by treating MSCs with 1 μg/mL BODIPY (493/503) (Life Technologies, Darmstadt, Germany) for 15 minutes, and nuclei were stained with 2 μM Hoechst-33342 for 5 minutes.

Chondrogenic differentiation was carried out in 96-well plates using the STEMPRO® Chondrogenesis Differentiation Kit (Gibco Life Technologies, Frankfurt, Germany) according to the manufacturer’s instructions. MSC spheroids were fixed with 4% paraformaldehyde solution before freezing at −20 °C and sectioning on a cryomicrotome. Sections were incubated in 1% Alcian Blue dissolved in 3% acetic acid solution for 30 minutes at room temperature, followed by washing steps using 0.1 M hydrochloric acid, PBS and deionizied water, respectively. Imaging was performed on a Keyence BioRevo9000 microscope (Keyence, Neu-Isenburg, Germany), and staining intensities were normalized to cell numbers and quantified using ImageJ software.

### DNA repair foci

MSCs were plated on coverslips and allowed to attach before treatment with 1800 ng/mL bleomycin for 4 hours. At the time points indicated in the Results section, cells were fixed with 4% paraformaldehyde. Cells were incubated with antibodies against γH2AX (1:100, Biolegend, London, UK), the catalytic subunit of phosphorylated DNA protein kinase (DNA-PK, 1:8000, Abcam, Cambridge, UK) and Rad51 protein (1:250, Cosmobio Co., Tokyo, Japan) overnight at 4 °C. After several washing steps, secondary antibodies (Alexa Fluor-568 goat anti-rabbit, 1:1000, Invitrogen, Darmstadt, Germany; Alexa Fluor-488 goat anti-mouse, 1:250, Invitrogen; DyLight 650 goat anti-chicken, 1:250, Thermo Fisher Scientific, Karlsruhe, Germany) were then added for 90 minutes at 4 °C. Cell nuclei were stained with 4’,6-diamidino-2-phenylindole (DAPI). For each condition and time point, 300 cells were automatically detected and assessed by an Axioplan-2 microscope (Zeiss, Jena, Germany) at 40x magnification using Metafer software (Metasystems, Altlussheim, Germany). Each experimental condition was analyzed in triplicate. Foci analysis on a single cell level was carried out using Matlab software (The MathWorks, Natick, Massachusetts, USA).

### Gene expression analysis

Cellular gene expression of MSC samples and HS68 fibroblasts was measured by a whole human genome microarray 4×44k (Agilent Technologies, Böblingen, Germany). RNA extraction was performed in log-phase cells using an RNeasy Mini Kit (Qiagen, Hilden, Germany). Data were extracted with the Agilent feature extraction software (version 9.1) and investigated. Statistical analysis was carried out by paired Student’s t-tests.

### Western blot analyses

MSCs and HS68 fibroblasts were treated with 400 or 1800 ng/mL bleomycin for 4 hours, and cells were harvested at 24 hours after completion of treatment. Samples containing 10 μg of protein from whole-cell lysates were run on a polyacrylamide gel before transfer to a polyvinylidene difluoride membrane (Millipore, Darmstadt, Germany). Membranes were incubated with a mouse monoclonal antibody against BLMH (1:100, Santa Cruz Biotechnology, Heidelberg, Germany) overnight at 4 °C, and β-actin was used as a loading control (1:2000, Cell Signaling Technology, Leiden, Netherlands). Blots were visualized using a horseradish peroxidase kit (Cell Signaling Technology).

## Additional Information

**How to cite this article**: Nicolay, N. H. *et al.* Mesenchymal stem cells are sensitive to bleomycin treatment. *Sci. Rep.* **6**, 26645; doi: 10.1038/srep26645 (2016).
